# Essential Updates 2024–2025: Surgical Strategy for Esophageal Cancer Toward a New Paradigm in the Era of Immunotherapy and Personalization

**DOI:** 10.1002/ags3.70197

**Published:** 2026-02-15

**Authors:** Shuichiro Oya, Koichi Yagi, Yoshifumi Baba

**Affiliations:** ^1^ Department of Gastrointestinal Surgery, Graduate School of Medicine The University of Tokyo Tokyo Japan

**Keywords:** artificial intelligence, esophageal neoplasms/surgery, frailty, immunotherapy, minimally invasive surgical procedures

## Abstract

Esophageal cancer surgery is evolving from technical standardization to a paradigm of personalized, strategy‐oriented care. Robotic‐assisted techniques and enhanced perioperative protocols have improved safety, but the field is increasingly shaped by three forces: integration of immune checkpoint inhibitors (ICIs), population aging, and the rise of intelligent technologies such as artificial intelligence (AI) and extended reality. Adjuvant nivolumab after neoadjuvant chemoradiotherapy (nCRT) remains the standard for residual disease, while other regimens such as durvalumab or dual checkpoint blockade have not demonstrated consistent survival benefit. Neoadjuvant ICI strategies, particularly camrelizumab plus chemotherapy in esophageal squamous cell cancer (ESCC), achieve high pathological response rates without increasing surgical morbidity, and pooled analyses confirm their feasibility. Immune‐related adverse events (irAEs) occur in approximately 20%–35% of patients but are usually manageable. Perioperative outcomes remain comparable to conventional regimens. Elderly and frail patients require individualized optimization. The integration of minimally invasive techniques, assessment of sarcopenia and nutritional risk, and adjustment of chemotherapy intensity have contributed to improved outcomes. Moreover, salvage surgery—once prohibitive—now offers meaningful long‐term survival when performed in high‐volume centers with specialized expertise. AI and machine learning are transforming risk stratification, intraoperative guidance, and surgical training. AI‐assisted video analytics and VR/AR simulators enhance skill acquisition, credentialing, and standardization. Future progress will depend on multicenter validation, prospective registries, and integration of oncologic, physiologic, and technological variables. Ultimately, the future of esophageal cancer surgery will be defined not only by technical precision but also by the surgeon's ability to leverage data‐driven innovation for personalized care.

## Graphical abstract

This review summarizes key advances from 2024 to 2025 that are reshaping esophageal cancer surgery toward a strategy‐oriented, personalized paradigm through the integration of immunotherapy, population aging, and intelligent technologies. Adjuvant nivolumab after neoadjuvant chemoradiotherapy remains the only perioperative approach with durable benefit, while emerging neoadjuvant immunochemotherapy, frailty‐informed decision‐making, and AI‐assisted support are redefining contemporary surgical strategy.

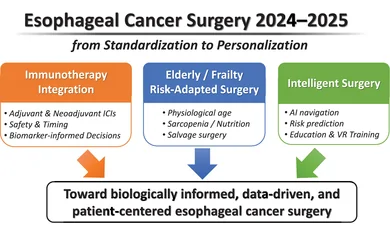

## 1 Introduction

### 1.1 From Standardization to Personalization in Esophageal Cancer Surgery

Esophageal cancer remains a highly lethal disease characterized by diverse histological diversity, regional variation, and heterogeneous treatment strategies, underscoring the need for reappraisal of contemporary surgical paradigms [1]. While robotic‐assisted techniques and advances in perioperative management have substantially improved surgical safety, the field is now being reshaped by three converging forces: the integration of immune checkpoint inhibitors (ICIs), a rapidly aging patient population, and the emergence of artificial intelligence (AI)‐and extended reality–assisted surgical technologies.

Against this backdrop, the role of the esophageal cancer surgeon is shifting from that of a technical operator to a strategic decision‐maker who must balance oncologic efficacy, physiologic reserve, and technological innovation to achieve truly personalized care. Accordingly, this review is organized around three key themes. First, we summarize recent advances in the perioperative ICI integration and their implications for surgical strategy. Second, we address surgical decision‐making in elderly and frail patients, in whom physiologic reserve, sarcopenia, and nutritional vulnerability critically influence the feasibility and benefit of multimodal treatment, including salvage and high‐risk surgery. Third, we review emerging applications of AI and extended reality that are transforming intraoperative navigation, risk prediction, and surgical education.

By synthesizing pivotal evidence published between 2024 and 2025, we propose a framework for individualized esophageal cancer surgery situated at the intersection of oncology, surgery, and data science.

## 2 Integration of Immunotherapy and Surgery: Redefining the Role of Resection

### 2.1 Adjuvant ICI Therapy

The phase III CheckMate 577 trial established adjuvant nivolumab as the standard for patients with esophageal or gastroesophageal junction (GEJ) cancer who had residual disease after neoadjuvant chemoradiotherapy (nCRT) and R0 resection, demonstrating a significant improvement in disease‐free survival (DFS) compared with placebo (22.4 vs. 11.0 months; hazard ratio [HR], 0.69), with manageable toxicity and approximately 10% treatment discontinuation [2]. This long‐term efficacy was sustained in the 2025 ASCO update, showing prolonged DFS (21.8 vs. 10.8 months; HR, 0.76), distant metastasis‐free survival (DMFS) (27.3 vs. 14.6 months; HR, 0.75), and a favorable 5‐year overall survival (OS) trend (46% vs. 41%; HR, 0.85) [3]. The benefit was more pronounced in esophageal squamous cell carcinoma (ESCC) and in tumors with a combined positive score (CPS) ≥ 1. Grade 3–4 AEs occurred in 14%, without new safety signals, and treatment initiation within 4–12 weeks postoperatively is recommended (Table 1).

**TABLE 1:** Major trials evaluating adjuvant immune checkpoint inhibitor therapy in esophageal and gastroesophageal junction cancer.

Trial/Year	Population/Histology	Neoadjuvant regimen	Arm A (Immunotherapy)	Arm B (Control)	Primaryendpoint	Key outcomes	Notes
CheckMate 577 [2] (Kelly 2021)	ESCC/EAC/GEJ with residual disease post‐nCRT, R0 resection (*n* = 794)	CROSS‐type CRT	Nivolumab 240 mg q2w for 16 week to 480 mg q4w up to 1 year	Placebo	DFS	DFS: 22.4 vs. 11.0 month (HR = 0.69)	Established the global standard for adjuvant ICI
CheckMate 577 2025 ASCO update [3] (Kelly 2025)	Same as above	CROSS‐type CRT	Nivolumab	Placebo	DFS	DFS: HR = 0.76 DMFS: HR = 0.75 5‐year OS: 46% vs. 41% (HR 0.85; not significant) Grade ≥ 3 TRAEs: ~14%	Long‐term data confirmed durable benefit
Durvalumab phase II [4] (Park 2022)	ESCC, post‐nCRT R0 resection (*n* = 86)	CRT (CROSS‐like)	Durvalumab 10 mg/kg q2w for 1 year	Placebo	DFS	DFS: HR = 1.18 (NS) OS: HR = 1.08 (NS) PD‐L1–positive subgroup: OS 94% vs. 64% (HR = 0.42)	No overall survival benefit; possible PD‐L1–related signal (different results from MATTERHORN [5])
VESTIGE [6] (Lordick 2025)	GE adenocarcinoma, ypN+ and/or R1 post‐nCT (*n* = 243)	nCT (FLOT, etc.)	Nivolumab 3 mg/kg q2w + Ipilimumab 1 mg/kg q6w for 1 year	Standard adjuvant chemotherapy	DFS	DFS: 11.4 vs. 20.8 month (HR = 1.55) Trial stopped early due to inferior outcomes	Intensified dual ICI was inferior to standard chemotherapy

Abbreviations: ASCO, American Society of Clinical Oncology; CRT, chemoradiotherapy; DFS, disease‐free survival; DMFS, distant metastasis‐free survival; OS, overall survival; EAC, esophageal adenocarcinoma; ESCC, esophageal squamous cell carcinoma; FLOT, fluorouracil, leucovorin, oxaliplatin, and docetaxel; GEJ, gastroesophageal junction; HR, hazard ratio; ICI, immune checkpoint inhibitor; MATTERHORN, phase III study of durvalumab plus FLOT as perioperative therapy for resectable gastric and gastroesophageal junction adenocarcinoma; mo, month(s); nCRT, neoadjuvant chemoradiotherapy; nCT, neoadjuvant chemotherapy; NS, not significant; PD‐L1, programmed death‐ligand 1; q2w/q4w/q6w, every 2/4/6 weeks; R0 resection, microscopically margin‐negative resection (no residual tumor); TRAE, treatment‐related adverse event; wk., week(s).

By contrast, a randomized phase II trial of adjuvant durvalumab in esophageal squamous cell carcinoma (ESCC) did not improve DFS or OS (HR 1.18 and 1.08, respectively) [4]. Exploratory analyses suggested a potential OS benefit in advantage in patients with Programmed death‐ligand 1(PD‐L1)–positive tumors (36‐month OS 94% vs. 64%; HR 0.42), whereas outcomes temded to be worse in PD‐L1–negative disease. These findings underscore the importance of biomarker‐driven selection in the adjuvant setting and contrast with the event‐free survival (EFS) benefit observed in the MATTERHORN trial of perioperative durvalumab plus chemotherapy in gastric and GEJ adenocarcinoma [5].

Similarly, the phase II VESTIGE trial comparing intensified adjuvant immunotherapy (nivolumab plus ipilimumab) with standard chemotherapy in high‐risk GEJ adenocarcinoma (ypN+ and/or R1) showed inferior DFS in the immunotherapy arm (11.4 vs. 20.8 months; HR 1.55), prompting early termination [6].

Taken together, adjuvant nivolumab after nCRT remains the only strategy with consistent and durable benefit in this setting. In East Asia, however, where chemotherapy‐based neoadjuvant regimens predominate for ESCC, the role of postoperative adjuvant immunotherapy remains less clearly defined due to the absence of confirmatory phase III evidence.

### 2.2 Neoadjuvant ICI Therapy

In East Asian populations, where ESCC predominates, neoadjuvant immunochemotherapy has progressed to phase III evaluation, although chemotherapy‐based regimens remain the backbone of neoadjuvant treatment. In Japan, the JCOG1109 study established preoperative docetaxel, cisplatin, and 5‐fluorouracil (DCF) as the standard neoadjuvant regimen [7]. By contrast, in Western countries—where adenocarcinoma of the esophagus and gastroesophageal junction (GEJ) is more prevalent—perioperative fluorouracil, leucovorin, oxaliplatin, and docetaxel(FLOT) demonstrated superiority over preoperative chemoradiotherapy(CRT) [8]. Against this background, ICIs have been explored in combination with chemotherapy or CRT (Table 2).

**TABLE 2:** Major trials evaluating neoadjuvant immune checkpoint inhibitor (ICI) therapy in esophageal cancer.

Trial/Year	Histology/Population	Regimen	Sample size	pCR (%)	MPR (%)	R0 Resection (%)	Grade ≥ 3 AEs (%)	Notes
Adenocarcinoma								
KEYNOTE 585 [9] (Shitara 2024)	Resectable Gastric/GEJ adenocarcinoma	Pembrolizumab + cisplatin‐based CT (main); Pembrolizumab + FLOT	main: *n* = 804 FLOT: *n* = 203	12.9 (main)	—	—	78	Improved pathological response but without a statistically significant survival benefit.
PERFECT [10] (van den Ende 2021)	Resectable EAC	Atezolizumab + CROSS regimen	*n* = 40	25	37.5	83	40 (irAE 0.8)	No survival benefit compared with matched controls.
Esophageal squamous cell carcinoma (ESCC)								
PALACE‐1 [11] (Li 2021)	Resectable ESCC	Pembrolizumab + CRT	*n* = 20	55.6	—	100	65	Feasible; one treatment‐related death occurred. No surgical delay.
PEN‐ICE [12] (Duan 2022)	Resectable ESCC	Pembrolizumab + CT	*n* = 18	46.2	69.2	100	27.8	Suggests a strong rationale for further randomized controlled trials.
TD‐NICE [14] (Yan 2022)	Resectable ESCC	Tislelizumab +CT	*n* = 45	40.0	57.5	80.5	42.2 (irAE 22.2)	Manageable irAEs; no treatment‐related deaths
Liu 2022, Phase II [15]	Resectable ESCC	Camrelizumab + CT	*n* = 60	29	47	—	—	Findings supported the subsequent phase III ESCORT‐NEO/NCCES01 trial.
ESCORT‐NEO/NCCES01 [16] (Qin 2024)	Resectable ESCC	Camrelizumab + CT (nab‐PTX + CDDP or PTX + CDDP)	*n* = 391	28.0 (nab‐PTX) 15.4 (PTX)	59.1 36.2	99.1 95.7	34.1 29.2	Higher pCR; EFS data immature.
JCOG1804E, FRONTiER [20] (Yoshii 2024)	Resectable ESCC	Nivolumab + FLOT	12	41.7	—	91.7	58	Japanese phase I trial; feasibility was the primary endpoint.

Abbreviations: AE, adverse event; CDDP, cisplatin; CRT, chemoradiotherapy; CT, chemotherapy; DCF, docetaxel, cisplatin, and 5‐fluorouracil; EAC, esophageal adenocarcinoma; EFS, event‐free survival; ESCC, esophageal squamous cell carcinoma; FLOT, 5‐fluorouracil, leucovorin, oxaliplatin, and docetaxel; GEJ, gastroesophageal junction; HR, hazard ratio; ICI, immune checkpoint inhibitor; irAE, immune‐related adverse event; mo, month(s); MPR, major pathological response; nab‐PTX, nanoparticle albumin‐bound paclitaxel; OS, overall survival; pCR, pathological complete response; PS, performance status; PTX, paclitaxel; R0 resection, microscopically margin‐negative resection (no residual tumor); wk., week(s).

#### 2.2.1 Adenocarcinoma

Evidence supporting neoadjuvant or perioperative ICI in adenocarcinoma remains inconsistent. The phase III KEYNOTE‐585 trial failed to show an improvement in EFS with perioperative pembrolizumab plus FLOT, despite a higher pathological complete response (pCR) rate [9].

Similarly, the PERFECT trial of atezolizumab combined with the CROSS regimen in 40 patients achieved a pCR rate of 25% but did not improve survival compared with matched nCRT cohorts, although exploratory biomarker analyses suggested predictive signatures [10]. While perioperative durvalumab plus FLOT has demonstrated benefit in Western gastric and GEJ adenocarcinoma, its applicability to esophageal adenocarcinoma remains uncertain.

#### 2.2.2 Esophageal Squamous Cell Carcinoma (ESCC)

In contrast, neoadjuvant immune checkpoint inhibition has shown more consistent activity in ESCC, particularly in East Asia. Early phase II studies of pembrolizumab‐based regimens reported encouraging pCR rates of approximately 45%–55% [11, 12]. More mature evidence subsequently emerged with next‐generation PD‐1 inhibitors: tislelizumab demonstrated a significant OS benefit in unresectable or metastatic ESCC in the RATIONALE‐306 trial [13] and achieved high pathological responses in the neoadjuvant TD‐NICE trial (MPR 72%, pCR 50%, R0 80.5%) [14] without treatment‐related surgical delays or deaths.

Evidence was further strengthened by camrelizumab. Following encouraging phase II results (pCR 39.2% and MPR 68.6%) [15], the subsequent phase III ESCORT‐NEO (NCCES01) trial (*n* = 391) demonstrated a significantly higher pCR rate with camrelizumab plus chemotherapy compared with chemotherapy alone (28.0% with nab‐paclitaxel/cisplatin vs. 15.4% with paclitaxel/cisplatin and 4.7% with chemotherapy alone), without excess surgical morbidity (34%–39%). These findings position camrelizumab‐based regimens as a potential neoadjuvant standard in China, although survival data remain immature [16].

#### 2.2.3 Integrated Evidence

Across histologies and treatment strategies, meta‐analyses consistently show that neoadjuvant immunochemoradiotherapy (nICRT) and immunochemotherapy (nICT) achieve high pCR and MPR rates with surgical feasibility comparable to conventional regimens [17, 18, 19]. A recent network meta‐analysis ranked camrelizumab plus chemotherapy as achieving the highest pCR/MPR, whereas pembrolizumab‐based regimens showed the most favorable objective response rate (ORR) and disease control rate (DCR) [19]. In Japan, early‐phase feasibility studies such as JCOG1804E (FRONTiER) are evaluating nivolumab with fluoropyrimidine‐based regimens in resectable esophageal cancer, with efficacy outcomes still immature [20].

### 2.3 Safety and Surgical Considerations

#### 2.3.1 Immune‐Related Adverse Events

Perioperative immune‐related adverse events (irAEs) occurred in approximately 20%–35% of patients receiving ICIs. In CheckMate 577, grade 3–4 treatment‐related adverse events (TRAEs) were reported in 14% of patients, without new safety signals [3].

In the neoadjuvant setting, pembrolizumab‐based regimens showed grade ≥ 3 AEs ranging from 27.8% with chemotherapy (PEN‐ICE) to 65% with CRT (PALACE‐1) [11, 12]. Tislelizumab (TD‐NICE) showed grade 3–4 TRAEs in 42% and irAEs in 22%, mostly low grade [14].

Meta‐analyses reported overall grade ≥ 3 AE rates of 20%–35% for neoadjuvant chemoimmunotherapy, comparable to conventional regimens and without increased surgical morbidity [21]. Common irAEs included rash, thyroid dysfunction, and pneumonitis, usually grade 1–2 and manageable. Pembrolizumab was associated with slightly fewer irAEs than other ICIs [19].

#### 2.3.2 Surgical Timing and Technical Issues

Across trials, surgery was typically scheduled 4–6 weeks after the last ICI dose [11, 12, 14, 16], resulting high R0 resection rates with minimal treatment disruption. Excessive delays may increase fibrosis, whereas shorter intervals risk unresolved irAEs or impaired wound healing.

Multiple studies have confirmed the feasibility of minimally invasive esophagectomy following nICT, with short‐term outcomes comparable to surgery alone or conventional nCRT. Recent real‐world and multicenter analyses demonstrated similar complication rates, without increased pneumonia, anastomotic leakage, or operative time [22, 23].

#### 2.3.3 Lymph Node Response and Future Perspectives

A meta‐analysis of 14 studies (*n* = 3212) demonstrated that complete lymph node regression and nodal downstaging were strongly associated with improved survival (OS HR 0.47; DFS HR 0.42) [24, 25]. These findings support future strategies aimed at tailoring lymphadenectomy based on pathological response.

### 2.4 Molecular and Biomarker‐Guided Personalization in the Era of Multimodal Therapy

Beyond patient‐related factors such as age and frailty, personalization in esophageal cancer surgery is increasingly informed by tumor biology and treatment response. PD‐L1 expression represents one of the earliest clinically validated factors for treatment stratification. In CheckMate 577, the benefit of adjuvant nivolumab was more pronounced in tumors with a PD‐L1 combined positive score (CPS) ≥ 1, whereas no benefit was observed in CPS < 1 [3]. More recently, circulating tumor DNA (ctDNA) has emerged as a promising marker of molecular residual disease, with postoperative ctDNA positivity strongly associated with an increasing recurrence risk [26, 27]. Collectively, these biomarkers support the integration of biologically informed, risk‐adapted surgical and perioperative strategies [3, 25, 26, 27].

### 2.5 Key Updates 2024–2025

Adjuvant nivolumab remains the only strategy with durable benefit after neoadjuvant chemoradiotherapy, whereas alternative adjuvant approaches have not demonstrated consistent efficacy. Neoadjuvant chemoimmunotherapy yields high pathological response rates without compromising surgical safety, and emerging biomarkers are increasingly enabling risk‐adapted perioperative decision‐making.

## 3 Surgical Strategy for Elderly and Frail Patients: Risk‐Adapted and Individualized Management

### 3.1 Physiologic Age, Frailty, and Treatment Tolerance

#### 3.1.1 Neoadjuvant Chemotherapy (NAC)

**FIGURE 1:**
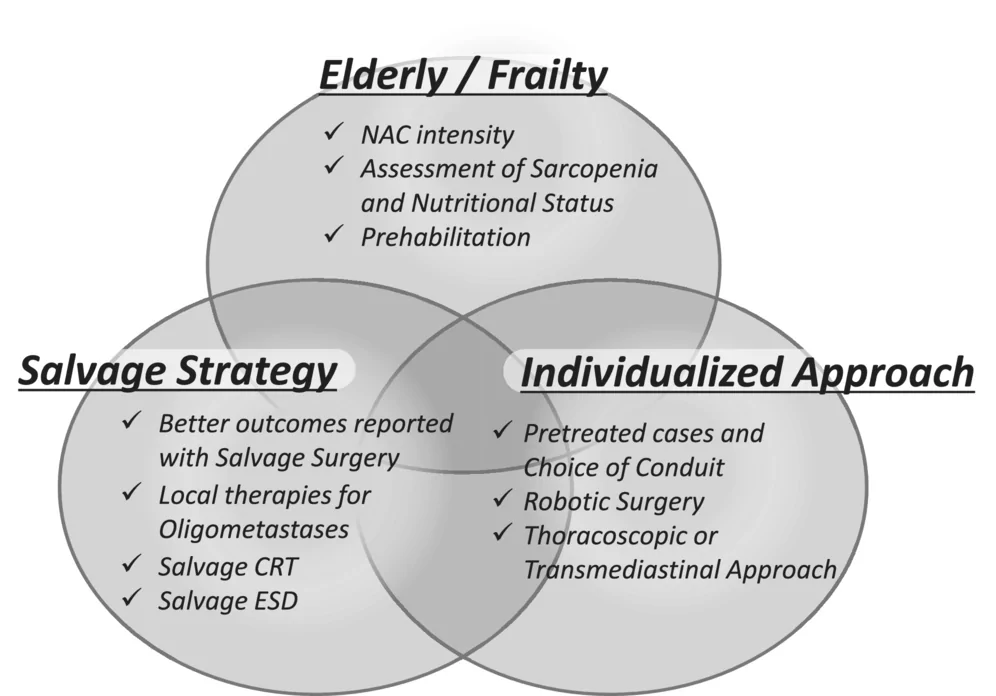
Conceptual framework for surgical decision‐making in challenging esophageal cancer cases. This Venn diagram illustrates three key domains influencing treatment strategies: Elderly and Frailty (assessment of sarcopenia, nutritional status, and prehabilitation to optimize neoadjuvant chemotherapy [NAC] intensity), Salvage Strategy (salvage surgery, chemoradiotherapy, and endoscopic approaches for oligometastases), and Individualized Approach (pretreatment status, conduit selection, thoracoscopic or transmediastinal techniques, and robotic surgery). The overlapping region highlights the importance of integrating patient condition, previous treatments, and surgical techniques to achieve individualized management.

The benefit of perioperative chemotherapy in elderly patients depends largely on baseline performance status (PS). A multicenter study showed that preoperative chemotherapy significantly improved 5‐year OS among patients with PS 0 (56.5% vs. 38.1%) but not in those with PS ≥ 1 [28]. Real‐world data indicate that dose reductions in subsequent NAC cycles are safe in patients aged ≥ 70 years without compromising long‐term survival [29] (Table 3 and Figure 1).

**TABLE 3:** Summary of key evidence (2024–2025) on surgical management for high‐risk or borderline esophageal cancer patients.

Subsection	Key evidence (Author, Year)	Population/setting	Main findings
Elderly/Frail	Yamashita 2024 [28]	Elderly, NAC	Survival benefit observed only in patients with PS 0.
	Skjoldbirk 2024 [29]	≥ 70 year, NAC	Dose reduction was safe without compromising survival.
	Sato 2025 [30]	ESCC, divided DCF	Benefit observed in sarcopenic patients.
	Luo 2024 [33] / Matsui 2025 [34] /Park 2024 [35]	Meta‐analyses	Meta‐analyses: Sarcopenia increased postoperative complications and was associated with poor OS and DFS.
	Qiu 2024 [37]	Pan‐cancer GNRI	GNRI was validated as a prognostic tool.
	An 2024 [38]/ Li 2025 [39]	Prehabilitation	Prehabilitation reduced pulmonary complications.
Salvage	Ozawa 2025 (SURGES) [45]	Salvage esophagectomy	3‐year OS approximately 49%; 90‐d mortality 3%. Severe complication rate 26%, leakage 13%, pneumonia 17%.
	Sugawara 2025 [46]		3‐year OS 45%–60% in patients with R0 resection; 90‐d mortality 2%–3%.
	Markar 2024 [48]	MIE/robotic salvage	Minimally invasive and robotic salvage surgery were feasible and enabled safer dissection.
	Apostolidis 2023 [49]	Oligometastatic	Local therapy improved overall survival (35 vs. 8 month).
	Cho 2025 [50]/ Katano 2025 [51]	Salvage CRT	Median OS approximately 30 months; dose‐dependent effect observed.
Individualized	Yasuda 2025 [52]	Jejunal graft	Jejunal graft was a reliable alternative to the stomach for reconstruction.
	Keating 2025 [44]	Network meta‐analysis	Conduits had comparable short‐term outcomes, with gastric conduit providing the best long‐term results.
	Takeuchi 2025 [54]	TE vs. OE (JCOG1409)	TE was non‐inferior to open esophagectomy (OE).
	Kooij 2025 [55]	RAMIE	RAMIE refined the thoracoscopic technique in TE
	Wang 2025 [56]	VATME vs. TE	VATME and TE showed similar outcomes and were feasible in high‐risk patients.
	Yagi 2025 [57]	RATME	RATME achieved overall survival comparable with transthoracic esophagectomy
	Fujita 2025 [58]	Robot‐assisted transcervical esophagectomy	Robot‐assisted transcervical esophagectomy demonstrated technical feasibility.

Abbreviations: CRT, chemoradiotherapy; d, day(s); DCF, docetaxel, cisplatin, and fluorouracil; DFS, disease‐free survival; ESCC, esophageal squamous cell carcinoma; GNRI, geriatric nutritional risk index; MIE, minimally invasive esophagectomy; mo, month(s); NAC, neoadjuvant chemotherapy; OE, open esophagectomy; OS, overall survival; PS, performance status; RAMIE, robot‐assisted minimally invasive esophagectomy; RATME, robot‐assisted transmediastinal esophagectomy; R0 resection, microscopically margin‐negative (complete) resection; TE, thoracoscopic esophagectomy; VATME, video‐assisted transmediastinal esophagectomy; y, year(s).

A biweekly divided‐dose DCF regimen failed to improve outcomes overall but offered survival benefit in sarcopenic patients with low psoas muscle index (PMI) [30]. PMI is commonly defined using sex‐specific cut‐off values (approximately < 6.3 cm^2^/m^2^ in men and < 3.9 cm^2^/m^2^ in women), derived from CT‐based sarcopenia definitions originally proposed by Prado et al. [31]. Consistently, a 2025 nationwide inpatient database study reported comparable perioperative morbidity between doublet and docetaxel‐based triplet NAC in older patients, supporting risk‐adapted regimen selection based on physiological reserve rather than chronological age alone [32]. Collectively, these findings suggest that reduced‐intensity regimens or upfront surgery may be preferable in patients with impaired physiological reserve rather than advanced chronological age alone.

#### 3.1.2 Sarcopenia and Nutritional Risk

Body composition and nutritional status are decisive prognostic factors in this population. Meta‐analyses published in 2024–2025 demonstrated that sarcopenia nearly doubles the risk of poor OS (HR ≈1.9) and DFS (HR ≈1.8), and increases postoperative complications by about 30% [33, 34, 35]. Sarcopenic obesity was associated with even worse survival outcomes [34].

Nutritional indices further complement these observations. The Geriatric Nutritional Risk Index (GNRI), originally proposed by Bouillanne et al. [36], is commonly categorized as > 98 (no risk), 92–98 (low risk), and < 92 (moderate to severe risk). Lower GNRI has been consistently associated with poor survival and increased postoperative morbidity, with a 2024 pan‐cancer analysis confirming its broad prognostic reliability [37].

#### 3.1.3 Prehabilitation and Supportive Strategies

A meta‐analysis of 16 upper gastrointestinal cancer studies found that prehabilitation reduced postoperative pneumonia (RR 0.71) and shortened hospital stay while improving functional recovery [38]. Likewise, a systematic review in esophagectomy showed lower pneumonia and pulmonary complication risks in prehabilitation cohorts (OR ≈0.48, 0.35), although evidence quality was limited and no significant differences were observed in major morbidity or mortality [39]. These findings support integrating structured exercise, nutrition, and multidisciplinary geriatric assessment into standard perioperative care.

#### 3.1.4 Surgical Outcomes

Accumulating multicenter and meta‐analytic evidence indicates that minimally invasive esophagectomy attenuates the excess perioperative risk traditionally observed in elderly patients, achieving outcomes comparable to younger cohorts, largely through reductions in pulmonary complications [28, 39]. These results align with landmark European multicenter and nationwide studies demonstrating perioperative safety, oncologic equivalence of minimally invasive approaches [40, 41], and benefits of treatment centralization in high‐volume centers [42].

In line with this paradigm, a 2024 nationwide propensity score‐matched study from Japan demonstrated that robot‐assisted minimally invasive esophagectomy achieved short‐term outcomes comparable to conventional minimally invasive approaches without increasing perioperative morbidity, supporting technical refinement rather than expansion of surgical indications [43]. Collectively, current evidence supports a risk‐adapted strategy in which chronological age alone should not preclude esophagectomy, provided that functional reserve, pulmonary risk, skeletal muscle mass, and nutritional status are appropriately assessed.

### 3.2 Salvage Surgery in Elderly and High‐Risk Patients

In elderly and physiologically frail patients, salvage surgery for locoregional recurrence represents one of the most challenging clinical scenarios, requiring careful balance between oncologic benefit and limited physiologic reserve. Available evidence indicates that meaningful benefit is confined to carefully selected patients, particularly those with isolated locoregional recurrence, absence of distant metastasis or T4 invasion, preserved performance status, and a realistic likelihood of achieving R0 resection [44].

Historically, salvage esophagectomy was associated with substantial morbidity and mortality; however, advances in perioperative management and centralization have markedly improved outcomes. The multicenter SURGES study reported overall complications of 46.6%, severe complications of 26.3%, anastomotic leakage in 12.8%, and pneumonia in 16.9%, with 30‐ and 90‐day mortality rates of 0% and 3.3%, respectively. Three‐year OS and PFS reached 48.9% and 40.0%, demonstrating meaningful long‐term benefit when R0 resection is achieved [45]. Consistently, a Japanese cohort reported 3‐year OS of 45%–60% in patients with successful R0 resection and 90‐day mortality of only 2%–3% [46], underscoring the importance of centralization and appropriate patient selection.

Technical advances have further contributed to improved outcomes. Salvage minimal invasive esophagectomy (MIE) after dCRT reduced overall complications (50% vs. 79%) and pneumonia (20% vs. 48.3%) compared with open surgery [47], and an observational cohort suggested better survival with MIE than open salvage surgery [48]. In parallel, local therapies has gained increasing relevance for limited‐volume or oligometastatic recurrence, with resection or stereotactic radiotherapy extended median OS to 35.2 months versus 7.8 months without local treatment (HR 0.21) [49].

Salvage chemoradiation remains a relevant alternative, with a median OS around 30 months; higher radiation doses (> 60 Gy) and longer recurrence‐free intervals predict improved outcomes [50, 51].

Collectively, these data redefine salvage therapy as a realistic curative option in selected elderly and high‐risk patients, with outcomes approaching those of planned surgery when careful resection and modern perioperative strategies are applied.

### 3.3 Individualized Surgical Approaches After Prior Treatments

#### 3.3.1 Reconstruction in the Absence of the Stomach

When the stomach cannot serve as a conduit, the jejunum has become a dependable alternative. A 2025 Japanese multicenter study demonstrated favorable safety and functional outcomes with free jejunal grafts using microvascular anastomosis, confirming feasibility in high‐volume centers [52]. A recent systematic review and network meta‐analysis reported comparable short‐term outcomes among gastric, colonic, and jejunal conduits, whereas long‐term survival remained superior with gastric reconstruction, underscoring the need for individualized organ selection [53].

#### 3.3.2 Surgical Approach in High‐Risk Patients

In the interim analysis of the JCOG1409 trial, a randomized study of 300 patients with stage I–III thoracic esophageal cancer, thoracoscopic esophagectomy (TE) proved non‐inferior to open esophagectomy (OE) in overall survival, with better relapse‐free survival and respiratory outcomes, establishing TE as a contemporary standard [54]. Robot‐assisted minimally invasive esophagectomy (RAMIE) further refines the thoracoscopic technique by enhancing visualization and instrument precision, thereby reducing pulmonary complications and accelerating recovery while maintaining oncologic equivalence [55].

For patients with impaired pulmonary function or multiple comorbidities, non‐transthoracic approaches provide less invasive alternatives. A 2025 meta‐analysis found no significant differences in overall complications or survival between transmediastinal esophagectomy (TME) and TE, supporting feasibility in selected patients [56]. Subsequent studies confirmed the feasibility of robot‐assisted non‐transthoracic approaches, with robot‐assisted TME achieving long‐term survival comparable to transthoracic surgery in ESCC [57], and robot‐assisted transcervical esophagectomy showing favorable short‐term outcomes with acceptable morbidity [58].

Although TE remains the gold standard when extensive lymphadenectomy is required, these alternative approaches broaden surgical options for high‐risk patients. Collectively, these advances highlight a shift from procedure‐centered decision‐making toward patient‐centered, risk‐adapted surgical strategies.

### 3.4 Key Updates 2024–2025

Physiologic age, sarcopenia, and nutritional status—rather than chronological age—are the principal determinants of treatment tolerance and outcomes in esophageal cancer surgery. Risk‐adapted and minimally invasive strategies, including carefully selected salvage surgery, have expanded therapeutic options for elderly and frail patients when delivered in experienced, high‐volume centers.

## 4 Technological Innovations and the Rise of Intelligent Surgery

### 4.1 AI‐Assisted Intraoperative Navigation

**FIGURE 2:**
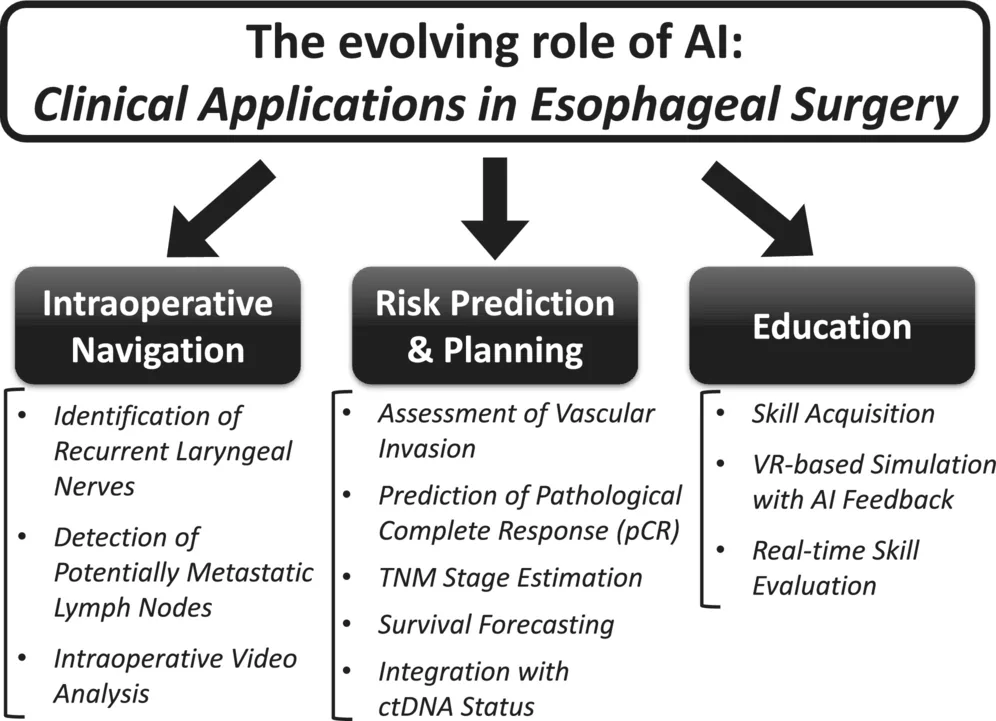
Clinical applications of artificial intelligence in esophageal surgery. This schematic illustrates three key domains in which artificial intelligence (AI) technologies are being applied: Intraoperative Navigation (identification of the recurrent laryngeal nerves, detection of potentially metastatic lymph nodes, and intraoperative video analysis), Risk Prediction and Planning (assessment of vascular invasion, prediction of pathological complete response [pCR], TNM stage estimation, survival prediction, and integration with circulating tumor DNA [ctDNA] status), and Education (skill acquisition, virtual reality–based simulation with AI feedback, and real‐time performance assessment). Together, these domains highlight the expanding role of AI in enhancing surgical safety, precision, and education in the field of esophageal surgery.

Recent advances in artificial intelligence (AI), building on foundational work in oncologic image interpretation, real‐time endoscopic diagnosis, and intraoperative workflow analysis [59, 60, 61, 62] have enabled data‐driven intraoperative navigation. Translational studies, particularly from East Asia, have demonstrated the feasibility of AI‐assisted navigation in esophageal cancer surgery (Table 4 and Figure 2).

**TABLE 4:** Applications of Artificial Intelligence and Machine Learning in Esophageal Cancer Surgery (2022–2025).

	Study (Author, Year)	Technology/Model	Cohort/Data	Main findings
Intraoperative navigation	Furube 2024 [63]	AI model trained on 120 RAMIE videos	RLN identification	IoU: 0.40 (right), 0.34 (left); trainees recognized RLN earlier (81.3% vs. 46.9%, *p* = 0.004)
	Furube 2025 [64]	Proof‐of‐concept traction detection	RAMIE intraoperative monitoring	Detected excessive traction on left RLN with 84.4% accuracy, before EMG changes
	Cizmic 2025 [65] (scoping review)	AI video analysis	117 studies	Identified applications in phase recognition, landmark detection, performance evaluation
Risk prediction & planning	Ma 2024 [67] (meta‐analysis)	Radiomics models	Multiple imaging datasets	LN metastasis prediction: a pooled AUC of approximately 0.74 (sensitivity 0.72)
	Huang 2024 [68]	Elastic‐net ML model	T1 ESCC	LN metastasis prediction AUC 0.803 versus 0.576 (guidelines)
	Wang 2024 [69]	CT radiomics (MRMR + LASSO)	Preop imaging	LN/vascular invasion prediction AUC approximately 0.89
	Zhang 2025 [70]	Vision‐Mamba DL model	nCIT patients	Predicted pCR after nCIT, AUC 0.83–0.92
	Li 2025 [71]	Multimodal radiomics nomogram	Imaging datasets	Predicted TNM stage, perineural invasion; AUC 0.72–0.90
	Winter 2024 [73]	Gradient boosting ML	552 esophagectomy patients	Predicted 90‐day mortality; superior AUROC/AUPRC versus IESG
	Zheng 2024 [74]	DeepSurv, DeepHit	Multicenter cohort	Recurrence/survival prediction; C‐index approximately 0.74
	Hofste 2022 [26]	ctDNA integration	Post‐nCRT patients	ctDNA positivity correlated with higher recurrence (HR 2.8) and worse OS (HR 2.9).
Educational applications	Kawashima 2024 [75]	VR robotic training	Simulation study	Improved robotic skill acquisition, faster completion, outcomes comparable to dry‐lab
	Wong 2025 [76]	Procedure‐specific simulator	RAMIE anastomosis	Validated realistic, risk‐free rehearsal
	Fuchs 2022 [77]	Delphi consensus	International experts	Proposed structured RAMIE pathway incl. VR and AI feedback
	Deo 2025 [78]	EASE framework	Suturing videos	Real‐time skill evaluation, granular performance metrics
	Nasriddinov 2024 [79]	Multimodal video/audio ML	Trainer–trainee interactions	Classified feedback with F1 approximately 0.82

Abbreviations: AI, artificial intelligence; AR, augmented reality; AUC, area under the curve; AUPRC, area under the precision—recall curve; AUROC, area under the receiver operating characteristic curve; ctDNA, circulating tumor DNA; DL, deep learning; EASE, End‐to‐end Assessment of Suturing Expertise; EMG, electromyography; ESCC, esophageal squamous cell carcinoma; HR, hazard ratio; IESG, International Esodata Study Group; IoU, intersection over union; LASSO, least absolute shrinkage and selection operator; LN, lymph node; ML, machine learning; MRMR, minimum redundancy maximum relevance; nCIT, neoadjuvant chemoimmunotherapy; nCRT, neoadjuvant chemoradiotherapy; OS, overall survival; pCR, pathological complete response; RAMIE, robot‐assisted minimally invasive esophagectomy; RLN, recurrent laryngeal nerve; TNM, tumor‐node‐metastasis staging; VR, virtual reality.

AI‐assisted systems now support identification of critical structures such as the recurrent laryngeal nerve (RLN). A model trained on 120 RAMIE videos achieved intersection over union (IoU) scores of 0.40 for the right and 0.34 for the left RLN, while trainees using the system recognized the right RLN earlier than controls (81.3% vs. 46.9%, *p* = 0.004) [63]. A subsequent proof‐of‐concept study showed that AI‐based detection of excessive traction on the left RLN reached an accuracy of 84.4%, allowing corrective action before changes in functional nerve monitoring occurred [64].

In parallel, AI‐driven intraoperative video analysis enables real‐time recognition of anatomical landmarks and surgical phases, enhancing procedural safety and consistency [65]. Collectively, these developments suggest that AI may reduce nerve injury, optimize lymphadenectomy, and promote procedural standardization, aligning with early concepts of intelligent surgery [66].

### 4.2 Machine Learning for Risk Prediction and Surgical Planning

Radiomics‐based AI is likewise contributing to surgical planning. A 2024 meta‐analysis reported moderate diagnostic performance for predicting lymph node metastasis (pooled AUC ≈0.74, sensitivity 0.72) [67]. In T1 ESCC, an elastic‐net machine learning model outperformed guideline‐based prediction (AUC 0.803 vs. 0.576) [68].

More advanced approaches have integrated feature selection and deep learning. Enhanced CT features selected by minimum redundancy maximum relevance (MRMR) and least absolute shrinkage and selection operator (LASSO) achieved an AUC of nearly 0.89 for predicting lymphovascular invasion [69], while deep‐learning models predicted pathologic complete response (pCR) after nICT with AUCs of 0.83–0.92 [70]. A radiomics nomogram combining multimodal imaging further demonstrated strong discrimination for predicting TNM stage and perineural invasion, with AUCs ranging from 0.72 to 0.90 [71].

These data align with Western platform‐based strategies that emphasizing multimodal integration over isolated predictors [72]. Gradient boosting models showed superior AUROC and AUPRC for 90‐day mortality compared with established risk scores [73], and deep neural networks modestly outperformed Cox models for recurrence and survival prediction [74]. Integration of ctDNA with ML‐based analytics may further refine perioperative risk stratification [26].

### 4.3 Educational Applications and Skill Assessment

Building on early European work on surgical phase recognition and intraoperative workflow analysis [62], AI‐driven video analysis frameworks are increasingly applied to surgical education and objective skill assessment. Automated phase recognition and anatomic landmark detection now enable standardized evaluation of technical performance in robotic esophagectomy [65, 69], facilitating reproducible assessment across trainees and institutions.

The integration of virtual and augmented reality (VR/AR) platforms with AI is further transforming surgical training. VR‐based programs have been shown to improve robotic skill acquisition and reduced task completion time compared with non‐training cohorts [75], while procedure‐specific simulators for robotic esophagectomy have been validated as realistic rehearsal tools [76]. An international Delphi consensus defined RAMIE training pathways [77], emphasizing simulation and AI‐driven feedback systems [77].

Beyond navigation support, AI is increasingly applied to granular performance assessment. The EASE framework enables real‐time evaluation of suturing performance [78], while multimodal models integrating video and audio data accurately categorize trainer feedback, achieving F1 scores of approximately 0.82 [79].

Collectively, these technologies support standardized training, credentialing, and outcome evaluation across varying levels of surgical expertise. The remaining challenge include translating algorithmic performance into sustained clinical benefit through transparent validation, interoperability, and effective surgeon–AI collaboration.

### 4.4 Key Updates 2024–2025

AI‐assisted technologies are increasingly integrated into intraoperative navigation, risk prediction, and surgical education. Recent advances indicate their potential to enhance safety, procedural standardization, and training in esophageal cancer surgery.

## 5 Conclusion: Toward a Strategy‐Oriented and Biologically Informed Surgery

The role of the esophageal cancer surgeon is evolving from a purely technical operator to a strategic decision‐maker navigating increasingly complex multimodal therapies. With rapid advances in systemic treatments, immuno‐oncology, and real‐time diagnostics, surgical strategy is no longer defined solely by operative skill, but by the capacity to appropriately select, time, and integrate surgery within multidisciplinary treatment pathways. This shift requires the incorporation of tumor biology, patient frailty assessment, treatment response, and emerging technologies such as AI and intraoperative imaging.

Personalized surgery relies on comprehensive preoperative evaluation integrating genomics, ctDNA, radiomics, and structured frailty indices. Equally important is the implementation of dynamic risk modeling and intraoperative decision‐support systems that enable surgical plans to adapt in real time as new information emerges. Minimally invasive, function‐preserving procedures must be tailored to individual patient profiles, while AI‐driven training and assessment platforms will play a central role in standardizing technique and maintaining safety across levels of surgical expertise.

Future progress will depend on prospective multicenter registries, standardized surgical metrics, and close multidisciplinary collaboration. Ultimately, the future of esophageal cancer surgery will be defined not only by technical precision but by the surgeon's ability to synthesize biological insight, data intelligence, and clinical judgment to deliver truly personalized care.

## Author Contributions

**Shuichiro Oya:** conceptualization, writing – original draft, writing – review and editing, visualization. **Koichi Yagi:** writing – review and editing, conceptualization, supervision. **Yoshifumi Baba:** writing – review and editing, conceptualization, supervision.

## Funding

The authors have nothing to report.

## Conflicts of Interest

Author Y.B. serves on the Editorial Board of *Annals of Gastroenterological Surgery*. Y.B. has received lecture honoraria from Miyarisan Pharmaceutical Co. Ltd., Ono Pharmaceutical Co. Ltd., Bristol Myers Squibb Co. Ltd., and MSD Co. Ltd.; however, none of these companies provided financial support for the preparation of this manuscript. The funding source had no role in the design, practice or analysis of this study.
